# Cold-induced anaphylaxis: new insights into clinical and genetic characteristics

**DOI:** 10.3389/fimmu.2025.1558284

**Published:** 2025-02-21

**Authors:** Mojca Bizjak, Peter Korošec, Mitja Košnik, Julij Šelb, Urška Bidovec-Stojkovič, Manca Svetina, Samo Zver, Dejan Dinevski, Matija Rijavec

**Affiliations:** ^1^ University Clinic of Respiratory and Allergic Diseases Golnik, Golnik, Slovenia; ^2^ Faculty of Medicine, University of Maribor, Maribor, Slovenia; ^3^ Faculty of Pharmacy, University of Ljubljana, Ljubljana, Slovenia; ^4^ Faculty of Medicine, University of Ljubljana, Ljubljana, Slovenia; ^5^ Biotechnical Faculty, University of Ljubljana, Ljubljana, Slovenia; ^6^ Hematology Department, University Medical Centre Ljubljana, Ljubljana, Slovenia

**Keywords:** anaphylaxis, cold urticaria, hereditary α-tryptasemia, KIT p.D816V, mast cell, total IgE, tryptase

## Abstract

**Introduction:**

The pathogenesis of cold urticaria (ColdU) and cold-induced anaphylaxis (ColdA) remains poorly understood, and ColdA is underrepresented in anaphylaxis literature. Laboratory features to guide management are largely unknown. This study evaluated basal serum tryptase (BST) and total immunoglobulin E (IgE) levels in ColdU and ColdA, their associations with clinical features, and the utility of testing for the *KIT* p.D816V variant in blood leukocytes and hereditary α-tryptasemia (HαT).

**Methods:**

Ninety-two adults with ColdU were enrolled. ColdA was defined as a reaction involving skin and/or visible mucosal tissue with cardiovascular, respiratory, or gastrointestinal manifestations. Evaluations included patient history, standard cold stimulation testing (sCST) using an ice cube and TempTest^®^, and laboratory tests.

**Results:**

ColdA was diagnosed in 35.9% of patients. ColdU phenotypes based on sCST included typical ColdU (52.2%), localized cold-reflex urticaria (5.4%), and ColdU with negative sCST (42.4%). Negative sCST, compared to typical ColdU, was associated with fewer ColdA cases (*p* = 0.004) but more spontaneous wheals (*p* < 0.001). ColdA patients more frequently exhibited generalized wheals (*p* = 0.047), skin angioedema (*p* = 0.007), oropharyngeal/laryngeal manifestations (*p* < 0.001), and itchy earlobes (*p* = 0.002) than non-ColdA patients. Elevated BST levels (>11.4 ng/mL) in 9.8% of patients were attributed to *KIT* p.D816V and/or HαT. *KIT* p.D816V was detected in 6.6% of ColdU and 6.3% of ColdA patients. HαT prevalence was higher in ColdU (10.9%) and ColdA (15.2%) than the general population (estimated at 5.7%; *p* = 0.041 and *p* = 0.038). Total IgE levels were significantly higher in ColdA than non-ColdA (*p* = 0.021).

**Discussion:**

This study confirmed clinical features linked to ColdA previously identified by the multicenter COLD-CE study, including generalized wheals, skin angioedema, oropharyngeal/laryngeal manifestations, and itchy earlobes. We identified new high-risk features. ColdA is more frequently associated with typical ColdU than with ColdU with negative sCST, the latter being linked to spontaneous wheals. ColdA is additionally associated with higher total IgE levels. Furthermore, patients with ColdU and ColdA exhibit higher prevalence of *KIT* p.D816V and HαT compared to general population data, a finding not previously reported. Further research is needed to explore their clinical implications.

## 1 Introduction

Cold-induced anaphylaxis (**ColdA**) is a potentially life-threatening systemic reaction triggered by cooling in patients with cold urticaria (**ColdU**) (1–3). It requires the urgent administration of adrenaline but is often underrecognized and undertreated (1, 4). The condition remains poorly understood, with no universally accepted definition and limited insight into its underlying mechanisms (1, 5). Additionally, literature on anaphylaxis frequently overlooks ColdA (1).

The clinical evaluation of patients with ColdU and ColdA presents significant challenges. Standard cold stimulation testing (**sCST**) on the volar forearm, which involves a 5-minute test using an ice cube melting in a small amount of water and/or a TempTest^®^ device with a 4–44°C electrode, often produces negative results (2, 6). In the large multicenter COLD-CE study, sCST was negative in 25% (139/551) of enrolled patients (2). The following phenotypes of ColdU can be diagnosed based on sCST: typical ColdU (i.e., whealing directly over the stimulated area), cold-reflex urticaria (i.e., papular wheals adjacent to the stimulated area) and ColdU with negative sCST (i.e., no whealing within 10 minutes). To further define ColdU with negative sCST, alternative provocation methods are required, such as total body cooling (5, 7). While ColdA has been reported in over a third of patients with typical ColdU (1, 2), its prevalence in other ColdU subtypes remains unknown (5, 8).

Anaphylaxis results from the sudden systemic release of mediators from mast cells (**MCs**) and basophils (9). MCs are distributed throughout vascularized tissues (10), especially in the skin, airways, and gastrointestinal tract (11). Basophils circulate in the blood. The release of MC-derived mediators has been well-documented in ColdU (12–18).

Tryptase, predominantly expressed by MCs, exists in four isoforms, with α and β being the most prevalent (19). Pro-α- and β-tryptases, which are constitutively secreted into the serum, account for the majority of measured basal serum tryptase (**BST**) levels in healthy individuals (20). Elevated BST levels can be observed in hereditary α-tryptasemia (**HαT**), clonal MC diseases, or chronic kidney disease (21). The upper limit of normal BST levels in individuals without HαT is 11.4 ng/mL (21–23), while the upper normal level for individuals with HαT is 15 ng/mL (24).

HαT is characterized by an increased number of α-tryptase-encoding copies at the *TPSAB1* locus (25). Its reported prevalence in the general populations of the US, UK, and EU is 5.7% (26). These individuals have increased levels of mature α/β tryptase heterotetramers (22, 27), which may increase endothelial cell permeability, as demonstrated *in vitro* (22), or induce vibration-triggered degranulation of skin MCs (27). It has been proposed that HαT may augment symptoms associated with clonal MC diseases (22, 28–31), but several questions remain regarding its clinical relevance (24, 28, 32).

The term clonal MC disease includes cutaneous mastocytosis, systemic mastocytosis (**SM**), and monoclonal MC activation syndrome (**MMAS**) (33). SM is diagnosed if the major and one minor criterion or at least three of four minor World Health Organization criteria are present. The major criterion is the presence of infiltrates of ≥15 aggregated MCs in sections obtained from the extracutaneous organ(s). The minor criteria include: (a) ≥25% spindle-shaped MCs in histological sections or ≥25% atypical MCs in a BM smear; (b) *KIT* p.D816V missense variant at codon 816 (***KIT* p.D816V**) in the BM, blood, or extracutaneous organ; (c) MCs in the BM, blood, or extracutaneous organ express one or more of CD2, CD25, or CD30; and (d) BST level persistently >20 ng/mL (29, 34, 35). MMAS includes cases with the presence of the *KIT* p.D816V and/or aberrant CD25 expression on MCs (36–38). Novel, ultrasensitive methods allow the detection of *KIT* p.D816V in blood leukocytes, with the ability to quantify as few as 0.001% of *KIT* p.D816V-encoding alleles (31, 39–42).

In the COLD-CE study, *Hymenoptera* venom-triggered anaphylaxis (**HVA**) was identified as a risk factor for ColdA (2). Severe HVA has been associated with clonal MC disease (31, 43), and severe manifestations of ColdU in a patient with a *KIT* p.D816V have been reported (44). Patients with ColdU were observed to have higher total immunoglobulin E (**IgE**) levels compared to those with chronic spontaneous urticaria (45). Therefore, this study aimed to evaluate BST and total IgE levels in patients with ColdU and ColdA, analyze their associations with clinical features, and determine whether testing for the *KIT* p.D816V in blood leukocytes and HαT is useful in ColdU and ColdA.

## 2 Materials and methods

### 2.1 Study design and participants

This cross-sectional study included consecutive adult patients with signs and symptoms of ColdU (≤12 months prior to enrollment) who were evaluated at the University Clinic of Respiratory and Allergic Diseases Golnik between May 2019 and December 2022. No patients were excluded from the study. Ethical approval was obtained from the National Medical Ethics Committee of Slovenia (KME0120-62/2019/4), and written informed consent was obtained from all participants. Demographic data and patient history were collected (**Table 1**), and a full-body examination for cutaneous mastocytosis lesions was conducted.

**Table 1:** Comparison of clinical characteristics between patients with ColdA and those without.

	Total *n* = 92	ColdA *n* = 33 (35.9)	Non-ColdA *n* = 59 (64.1)	*p-*value
Demographics and baseline clinical characteristics				
Age (years)^a^	40.4 ± 13.7	42.2 ± 14.0	39.4 ± 13.6	0.361
Female gender^b^	64 (69.6)	24 (72.7)	40 (67.8)	0.814
Duration of ColdU (months)^c^	60.0 (14.3−129.0)	72.0 (32.5−162.5)	36.0 (13.0−120.0)	0.065
Age at onset of ColdU (years)^c^	33.0 (20.0−42.0)	33.0 (18.0−45.5)	32.0 (20.0−41.0)	0.935
Pediatric onset of ColdU (<18 years)^b^	15 (16.3)	8 (24.2)	7 (11.9)	0.147
Positive family history of ColdU^b^	5 (5.4)	2 (6.1)	3 (5.1)	1.000
Maximal wheal duration (minutes)^c^	60 (30−120), *n* = 89	60 (30−90)	60 (30−120), *n* = 56	0.727
Cold-induced clinical signs and symptoms				
Generalized wheals^b^	37 (40.2)	18 (54.5)	19 (32.2)	0.047*
Skin angioedema, any location^b^	33 (35.9)	18 (54.5)	15 (25.4)	0.007**
Oropharyngeal/laryngeal manifestations^b^	25 (27.2)	18 (54.5)	7 (11.9)	<0.001***
Itchy earlobes^b^	41 (44.6)	22 (66.7)	19 (32.2)	0.002**
Fever or arthralgia^b^	0	0	0	^NA^
Loss of consciousness or dizziness/weakness (= ColdA^Cardio^)^b^	25 (27.2)	25 (75.8)	0	^NA^
Dyspnea^b^	26 (28.3)	26 (78.8)	0	^NA^
Crampy abdominal pain or vomiting^b^	2 (2.2)	2 (6.1)	0	^NA^
Triggers of ColdU				
Cold foods or drinks^b^	25 (27.2)	18 (54.5)	7 (11.9)	<0.001***
Whole-body water immersion^b^	67 (72.8)	30 (90.9)	37 (62.7)	0.003**
Cold air^b^	66 (71.7)	27 (81.8)	39 (66.1)	0.148
Transition cold outdoors to warm indoors^b^	47 (51.1)	19 (57.6)	28 (47.5)	0.390
Cold surfaces^b^	39 (42.4)	20 (60.6)	19 (32.2)	0.015*
Wind^b^	39 (42.4)	22 (66.7)	17 (28.8)	0.001**
Summer rain^b^	34 (37.0)	18 (54.5)	16 (27.1)	0.013*
ColdU phenotype based on sCST				
Typical ColdU^b^	48 (52.2)	25 (75.8)	23 (39.0)	0.001**
Localized cold-reflex urticaria^b^	5 (5.4)	0	5 (8.5)	0.156
ColdU with negative sCST^b^	39 (42.4)	8 (24.2)	31 (52.5)	0.009**
Comorbidities (medical diagnosis)				
Chronic spontaneous urticaria^b,d^	18 (19.6)	3 (9.1)	15 (25.4)	0.098
Atopic disease^b^	30 (32.6)	11 (33.3)	19 (32.2)	1.000
Systemic reaction to *Hymenoptera* venom^b^	6 (6.5)	3 (9.1)	3 (5.1)	0.663
Thyroid disease^b^	8 (8.7)	2 (6.1)	6 (10.2)	0.707
Connective tissue disease^b^	2 (2.2)	1 (3.0)	1 (1.7)	1.000

Categorical variables are presented as counts (percentages), while numerical variables are expressed as mean ± SD for normally distributed data and median (IQR) for non-normally distributed data. If data were not obtained for all patients, the number of patients is displayed as “n.” Statistical significance of differences between groups was assessed using the Student’s *t*-test (^a^), Fisher’s Exact test (^b^), and Mann-Whitney test (^c^). Statistically significant *p*-values are highlighted in bold. Significance levels are indicated by *(*p* < 0.05), **(*p* < 0.01), and ***(*p* < 0.001). ^d^ColdU was the predominant subtype of chronic urticaria as determined from patient history.

*ColdA*, cold-induced anaphylaxis; *ColdA^Cardio^*, cold-induced anaphylaxis with cardiac involvement; *ColdU*, cold urticaria; *NA*, not applicable; *non-ColdA*, absence of cold-induced anaphylaxis; *sCST*, standard cold stimulation testing.

### 2.2 Standard 5-minute cold stimulation testing on the volar forearm

The sCST was performed on the volar forearm using an ice cube melting in a small amount of water within a non-latex glove and a TempTest^®^ 4.0 device, which has a 4–44°C electrode. A 5-minute stimulation period was used. Skin responses were assessed 10 minutes post-stimulation. H_1_-antihistamines and systemic glucocorticoids were discontinued at least 3 and 7 days prior to testing, respectively (6).

### 2.3 Definition of clinical phenotypes

Typical ColdU, localized cold-reflex urticaria, and ColdU with negative sCST were defined in accordance with above described criteria. The diagnosis of ColdU with negative sCST was established based on a reliable medical history of reactivity to cold stimuli and patients’ photographs of cold-induced wheals and/or angioedema in real life. ColdA was defined as a reaction involving the skin and/or visible mucosal tissue, along with at least one additional systemic manifestation: (a) cardiovascular (syncope [loss of consciousness] or near syncope [dizziness, weakness]), (b) respiratory (difficulty breathing [dyspnea]), or (c) gastrointestinal (crampy abdominal pain or vomiting) (2). The term ColdA with cardiac involvement (**ColdA^Cardio^**) referred to reactions involving the skin and/or visible mucosal tissue accompanied by syncope or near syncope. Cold-induced oropharyngeal and laryngeal manifestations (i.e., swelling in the oral cavity, painful swallowing [odynophagia], or hoarse voice) were not regarded as part of the signs and symptoms of ColdA; data on these manifestations were reported separately.

### 2.4 Laboratory workup

Blood samples were collected during >24-hour symptom-free intervals and before sCST. The laboratory workup included: (a) BST level (*n* = 92) by ImmunoCAP 100 (Thermo Fisher Scientific, Uppsala); (b) total serum IgE level (*n* = 92) by Immulite 2000Xpi (Siemens); (c) C-reactive protein (**CRP**) level (*n* = 92) by Cobas 6000 (Roche); (d) specific IgE levels to honeybee (i1; *n* = 90) and wasp (i3; *n* = 91) by Immulite 2000Xpi (Siemens Healthcare Diagnostics, Erlangen); (e) blood *KIT* p.D816V analysis (*n* = 91); and (f) tryptase genotyping (*n* = 29).

#### 2.4.1 Blood KIT p.D816V assay

Genomic DNA was extracted from 400 µL of EDTA-containing whole blood samples using a QIAamp DNA Blood Mini Kit (Qiagen, Hilden, Germany), following the manufacturer’s instructions. The *KIT* c.2447A>T, p.D816V missense variant (p.Asp816Val) and quantification of allele burden were assayed with allele-specific quantitative PCR (**qPCR**) (31, 39, 46). The ABI 7500 Fast Real-Time PCR system and SDS 2.3 software (Thermo Fisher Scientific, Uppsala) were used.

#### 2.4.2 Tryptase genotyping

Genotyping of *TPSAB1* and *TPSB2* was performed using multiplex droplet digital PCR (**ddPCR**) in individuals with BST level ≥6 ng/mL (*n* = 29), as previously described (25, 47). No individual with HαT has ever been reported with BST level <6.0 ng/mL (39). A manual droplet generator (Bio-Rad), QX200 droplet reader (Bio-Rad), associated QX Manager software (Bio-Rad), and custom primers and probes targeting specifically α- and β-tryptase sequences, along with primers and probes targeting *AP3B1* or *AGO1* as a reference gene, were used. Patients were arranged into two groups: unaffected and HαT genotypes.

### 2.5 Hematological assessment

Patients testing positive for *KIT* p.D816V in blood leukocytes were referred to the University Medical Centre Ljubljana for hematological evaluation, including bone marrow biopsy and aspiration (33, 48).

### 2.6 Statistical analysis

IBM SPSS version 25 was used for statistical analysis. Descriptive statistics included frequencies and proportions for categorical variables; ranges, means with 95% confidence intervals (CI), and standard deviations (SD) for normally distributed numerical variables; and medians and interquartile ranges (IQR) for non-normally distributed numerical variables. A *p*-value <0.05 was considered statistically significant. Categorical variables were assessed by Fisher’s exact test. Numerical variables with normal distribution and those not normally distributed were analyzed using Student’s t-test and Mann-Whitney test, respectively. Prevalence of HαT was compared to the hypothetical prevalence of the parameter in the general population, acquired from the literature, using the exact binomial test.

## 3 Results

### 3.1 Demographic and baseline clinical characteristics

A total of 92 ColdU patients were enrolled, 69.6% of whom were female. Patient ages ranged from 18 to 73 years (mean: 40.4 years). Key clinical features are summarized in **Table 1**. Pediatric onset of ColdU and a positive family history of ColdU were identified in 16.3% and 5.4% of patients, respectively. The following ColdU phenotypes were diagnosed based on sCST: typical ColdU in 52.2% (*n* = 48), localized cold-reflex urticaria in 5.4% (*n* = 5), and ColdU with negative sCST in 42.4% (*n* = 39) of patients. ColdA was diagnosed in 35.9% (*n* = 33) of patients, including ColdA^Cardio^ in 27.2% (*n* = 25). Concomitant chronic spontaneous urticaria was diagnosed in 19.6% (*n* = 18) of patients, with ColdU being the predominant subtype of chronic urticaria in these cases. Cold-induced oropharyngeal and laryngeal manifestations were reported by 27.2% (*n* = 25) of patients (**Table 1**).

### 3.2 Triggers of ColdU and ColdA

Patients reported the following triggers of ColdU (i.e., wheals and/or angioedema): cold foods or drinks (27.2%), whole-body water immersion (72.8%), cold air (71.7%), transitioning from cold outdoors to warm indoors (51.1%), contact with cold surfaces (42.4%), wind (42.4%), and summer rain (37.0%) (**Table 1**). ColdA was triggered by whole-body water immersion (*n* = 27), cold air (*n* = 8), or cold drinks (*n* = 2). Compared to non-ColdA patients, ColdA patients more frequently reported the following triggers of their wheals and/or angioedema: cold foods or drinks (*p* < 0.001), whole-body water immersion (*p* = 0.003), contact with cold surfaces (*p* = 0.015), wind (*p* = 0.001), and summer rain (*p* = 0.013) (**Table 1**).

### 3.3 Clinical features associated with ColdA

Patients with ColdA had a higher frequency of generalized wheals (*p* = 0.047), skin angioedema (*p* = 0.007), oropharyngeal/laryngeal manifestations (*p* < 0.001), itchy earlobes (*p* = 0.002), and typical ColdU (*p* = 0.001) compared to those without ColdA (**Table 1**). Patients with ColdA^Cardio^ had a higher frequency of generalized wheals (64.0% [16/25] vs. 31.3% [21/67], *p* = 0.008), skin angioedema (56.0% [14/25] vs. 28.4% [19/67], *p* = 0.026), oropharyngeal/laryngeal manifestations (52.0% [13/25] vs. 17.9% [12/67], *p* = 0.003), itchy earlobes (72.0% [18/25] vs. 34.3% [23/67], *p* = 0.002), and typical ColdU (80.0% [20/25] vs. 41.8% [28/67], *p* = 0.002) compared to those without.

### 3.4 Elevated BST levels were attributed to *KIT* p.D816V and/or HαT

**Figure 1:**
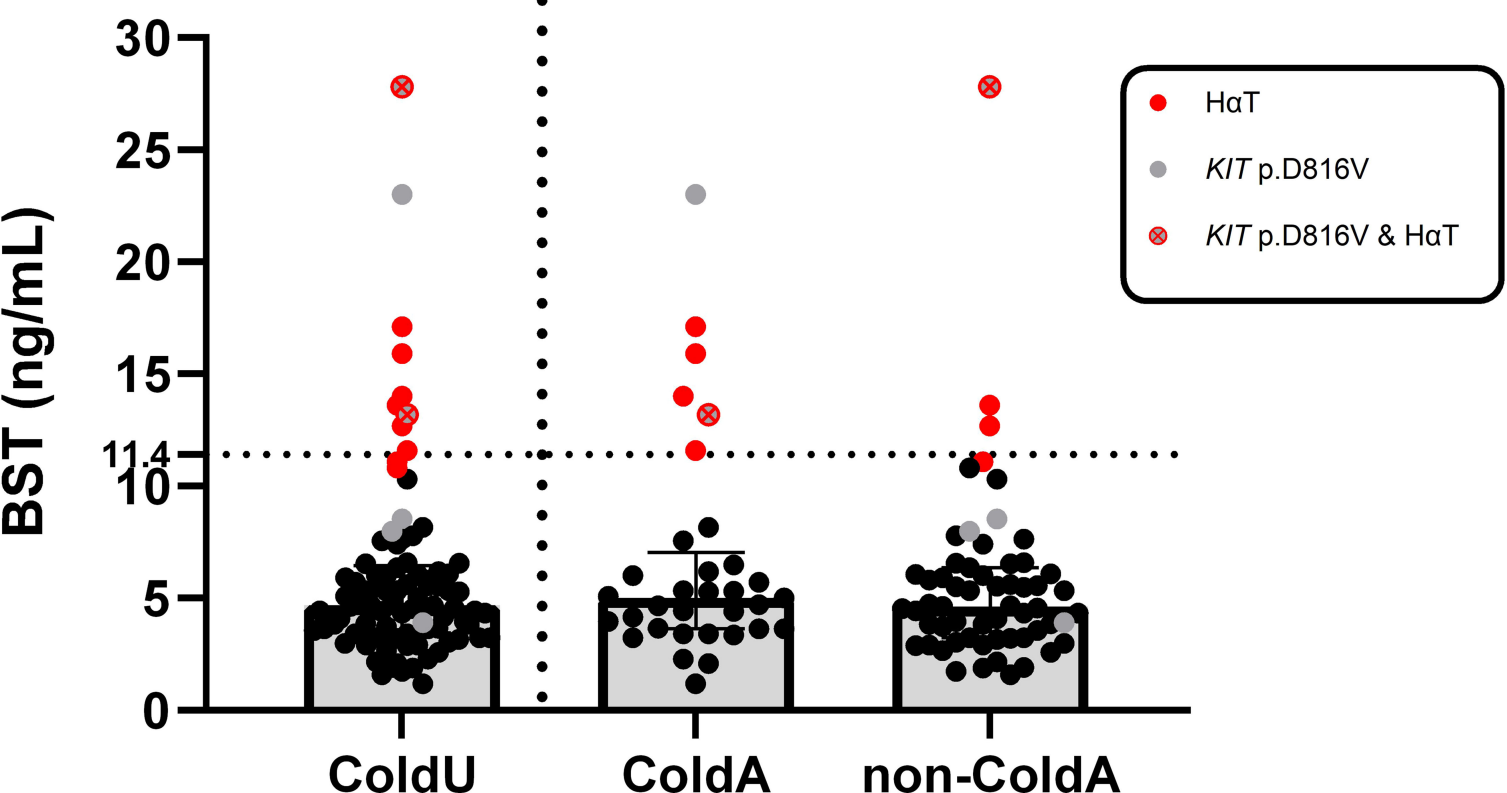
Relationship between BST levels and the presence of HαT and *KIT* p.D816V. The BST levels measured in ColdU patients ranged from 1.19 to 27.80 ng/mL. Elevated BST levels (>11.4 ng/mL) were identified in 9 ColdU patients, with elevations attributed to either HαT (*n* = 8, indicated by red circles) or the presence of *KIT* p.D816V (*n* = 3, represented by grey circles). Two patients had both conditions (marked by crossed red and grey circles). Three patients positive for *KIT* p.D816V demonstrated BST levels ≤11.4 ng/mL. *BST*, basal serum tryptase; *ColdA*, cold-induced anaphylaxis; *ColdU*, cold urticaria; *HαT*, hereditary α-tryptasemia; *KIT p.D816V*, *KIT* missense variant at codon 816; *non-ColdA*, absence of cold-induced anaphylaxis.

BST levels ranged from 1.19 to 27.80 ng/mL (median: 4.70 ng/mL). Elevated BST levels (>11.4 ng/mL) were identified in 9.8% (9/92) of ColdU patients (**Table 2**) and attributed to *KIT* p.D816V (*n* = 3) or HαT (*n* = 8) (**Figure 1**). Two patients had both *KIT* p.D816V and HαT (**Figure 1**, **Table 2**). The prevalence of elevated BST levels was higher in ColdA patients compared to non-ColdA patients, with the difference approaching statistical significance (18.2% vs. 5.1%, *p* = 0.065; **Table 2**).

**Table 2:** Comparison of laboratory characteristics between patients with ColdA and those without.

	Total *n* = 92	ColdA *n* = 33 (35.9)	Non-ColdA *n* = 59 (64.1)	*p-*value
BST				
BST level (ng/mL)^a^	4.70 (3.46−6.45)	5.02 (3.64−7.03)	4.63 (3.23−6.37)	0.476
Elevated BST level (>11.4 ng/mL)^b^	9 (9.8)	6 (18.2)	3 (5.1)	0.065
Elevated BST level (>15.0 ng/mL)^b^	4 (4.3)	3 (9.1)	1 (1.7)	0.130
Genetic tests				
*KIT p.D816V* ^b^	6 (6.6), *n* = 91	2 (6.3), *n* = 32	4 (6.8)	1.000
HαT^b^	10 (10.9)	5 (15.2)	5 (8.5)	0.486
*KIT p.D816V* and HαT^b^	2 (2.2), *n* = 91	1 (3.1), *n* = 32	1 (1.7)	1.000
*KIT p.D816V* or HαT^b^	14 (15.4), *n* = 91	6 (18.8), *n* = 32	8 (13.6)	0.552
Total serum IgE				
Total serum IgE level (IU/mL)^a^	96.0 (31.0−212.3)	113.0 (43.0−389.0)	63.0 (24.0−164.0)	0.021*
Total serum IgE level ≥100 IU/mL^b^	43 (46.7)	18 (54.5)	25 (42.4)	0.284
High total serum IgE level (≥75^th^ percentile; ≥212.3 IU/mL)^b^	23 (25.0)	13 (39.4)	10 (16.9)	0.024*
*Hymenoptera* sensitization				
Wasp sensitized (sIgE >0.35 IU/mL)^b^	37 (40.7), *n* = 91	13 (40.6), *n* = 32	24 (40.7)	1.000
Honeybee sensitized (sIgE >0.35 IU/mL)^b^	23 (25.6), *n* = 90	6 (19.4), *n* = 31	17 (28.8)	0.447
Wasp or honeybee sensitized^b^	46 (50.5), *n* = 91	15 (46.9), *n* = 32	31 (52.5)	0.664

Categorical variables are presented as counts (percentages), while numerical variables are expressed as median (IQR). If data were not obtained for all patients, the number of patients is displayed as “n”.

Statistical significance of differences between groups was assessed using the Mann-Whitney test (^a^) and Fisher’s Exact test (^b^). Statistically significant *p*-values are highlighted in bold. Significance levels are indicated by *(*p* < 0.05), **(*p* < 0.01), and ***(*p* < 0.001).

*BST*, basal serum tryptase; *ColdA*, cold-induced anaphylaxis; *HαT*, hereditary α-tryptasemia; *IgE*, immunoglobulin E; *KIT p.D816V*, KIT p.D816V missense variant at codon 816 detected in blood leukocytes; *non-ColdA*, absence of cold-induced anaphylaxis; *sIgE*, specific immunoglobulin E antibodies.

### 3.5 *KIT* p.D816V was detected in ColdU and ColdA

Analysis revealed that *KIT* p.D816V was present in 6.6% (6/91) of all ColdU patients, 4.2% (2/48) of typical ColdU patients, and 6.3% (2/32) of ColdA patients (**Table 2**, **Supplementary Tables S1**, **S2**). None of the enrolled patients had clinical signs of cutaneous mastocytosis. One patient fulfilled the diagnostic criteria for SM, four met the criteria for MMAS, and one remained unclassified due to declining a bone marrow biopsy (**Table 3**). BST levels for individual patients with *KIT* p.D816V are detailed in **Table 3**. Patients with this variant had higher BST levels compared to individuals without it (median [IQR]: 10.87 [7.90−24.20] vs. 4.56 [3.39−6.04] ng/mL, *p* < 0.001). Interestingly, half of the patients with *KIT* p.D816V had elevated BST levels, while the other half had normal levels. No statistically significant associations were found between the presence of *KIT* p.D816V and the clinical characteristics of ColdU or ColdA. However, a trend was observed, with *KIT* p.D816V-positive patients experiencing disease onset at an older age compared to those without this variant (median [IQR]: 47.0 [28.3−54.0] vs. 33.0 [20.0−41.0] years, *p* = 0.074; **Supplementary Table S2**).

**Table 3:** Clinical and laboratory characteristics of patients with the *KIT* p.D816V variant.

	Patient #1	Patient #2	Patient #3	Patient #4	Patient #5	Patient #6
Diagnosis: SM or MMAS	MMAS	MMAS	SM	*Nd*	MMAS	MMAS
Age (years)	65	35	47	58	56	70
Gender	f	m	f	f	m	f
BM biopsy analysis						
Infiltrates of ≥15 MCs in aggregates^a*^	*Nr*	–	–	*Nd*	–	–
≥25% spindle-shaped MCs^b^	*Nr*	–	+	*Nd*	–	–
*KIT* p.D816V^b^	**+**	**+**	+	*Nd*	*Nd*	–
MCs with ≥1: CD2, CD25, CD30^b^	*Nr*	–	+	*Nd*	–	–
Blood analysis						
*KIT* p.D816V^b^	**+**	**+**	+	**+**	**+**	**+**
BST level persistently >20 ng/mL^b^	+	–	+	–	–	–
BST level (ng/mL)	23.00	8.53	27.80	13.20	7.99	3.90
*KIT* p.D816V allele burden (%)	0.415	0.027	0.086	0.001	0.001	0.001
HαT	–	–	+	+	–	Nd
ColdA phenotype						
ColdA	+	–	–	+	–	–
ColdA with cardiac involvement	–	–	–	+	–	–
**Systemic reactions to *Hymenoptera* venom** ^c^	–	–	+ (IV)	–	+ (I)	–

The BM sample from patient #1 was not representative; MCs were slightly multiplied but did not form aggregates, and a few MCs were spindle-shaped. Patient #4 declined BM biopsy.

SM is diagnosed if the major^a^ criterion and one minor^b^ criterion, or at least three of four minor^b^ criteria are present. MMAS is defined by the presence of one or two minor clonality criteria: the *KIT* p.D816V variant and/or aberrant CD25 expression on MCs. ^c^Reaction severity grades were assigned according to the Mueller grading system (grades I−IV).

*BM*, bone marrow; *BST*, basal serum tryptase; *ColdA*, cold-induced anaphylaxis; *ColdA^Cardio^*, cold-induced anaphylaxis with cardiac involvement; *f*, female; *HαT*, hereditary α-tryptasemia; *KIT* p.D816V*, KIT* missense variant at codon 816; *m*, male; *MCs*, mast cells; *MMAS*, monoclonal mast cell activation syndrome; *Nd*, not determined; *Nr*, not representative; *SM*, systemic mastocytosis; +, yes/positive; *-*, no/negative.

### 3.6 Prevalence of HαT in ColdU and ColdA was higher than in the general population

**Figure 2:**
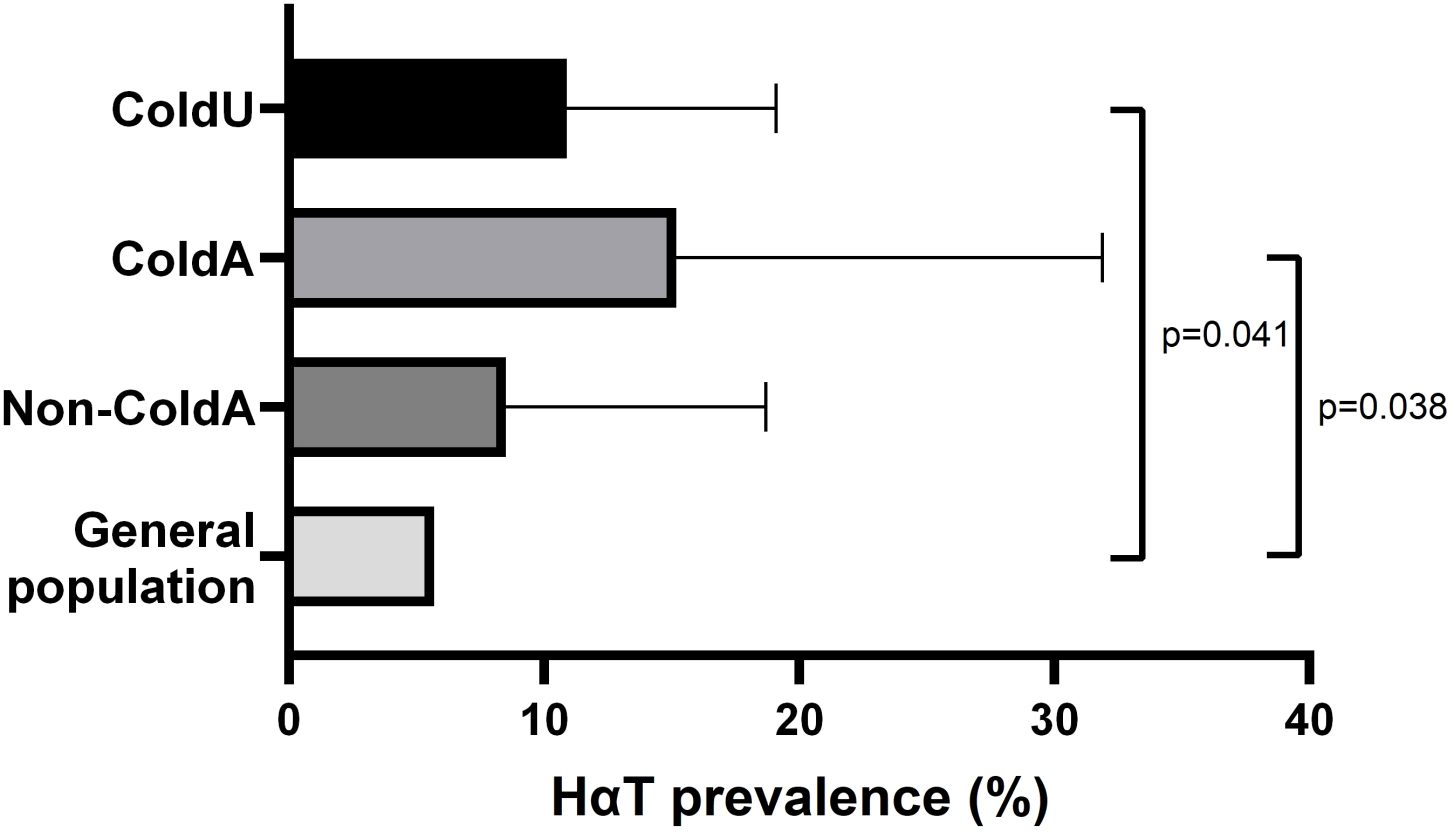
Prevalence of HαT. The prevalence of HαT was significantly higher in ColdU (10.9%) and ColdA (15.2%) compared to an estimated 5.7% prevalence in the general population, as determined using the exact binomial test. *ColdA*, cold-induced anaphylaxis, *ColdU*, cold urticaria; *HαT*, hereditary α-tryptasemia; *non-ColdA*, absence of cold-induced anaphylaxis.

Tryptase genotyping identified a significantly higher prevalence of HαT in ColdU (10.9% [10/92]) compared to the estimated 5.7% prevalence in the general population (26) (*p* = 0.041). Similarly, the prevalence of HαT was higher in ColdA (15.2% [5/33]) than in the general population (*p* = 0.038) (**Table 2**, **Figure 2**). Among the HαT-positive patients, three (30%) had a duplication of the *TPSAB1* gene (genotype αα/β), while the remaining seven (70%) had the genotype αα/α (**Supplementary Table S1**). Patients with HαT had significantly higher BST levels than those without the condition (median [IQR]: 13.40 [11.48−16.20] vs. 4.50 [3.34−5.84] ng/mL, *p* < 0.001). Eighty percent of HαT-positive individuals had BST levels exceeding 11.4 ng/mL, and 30% had BST levels above 15.0 ng/mL. HαT was not associated with clinical parameters of ColdU or ColdA (**Supplementary Table S3**).

### 3.7 ColdA was associated with higher total serum IgE

**Figure 3:**
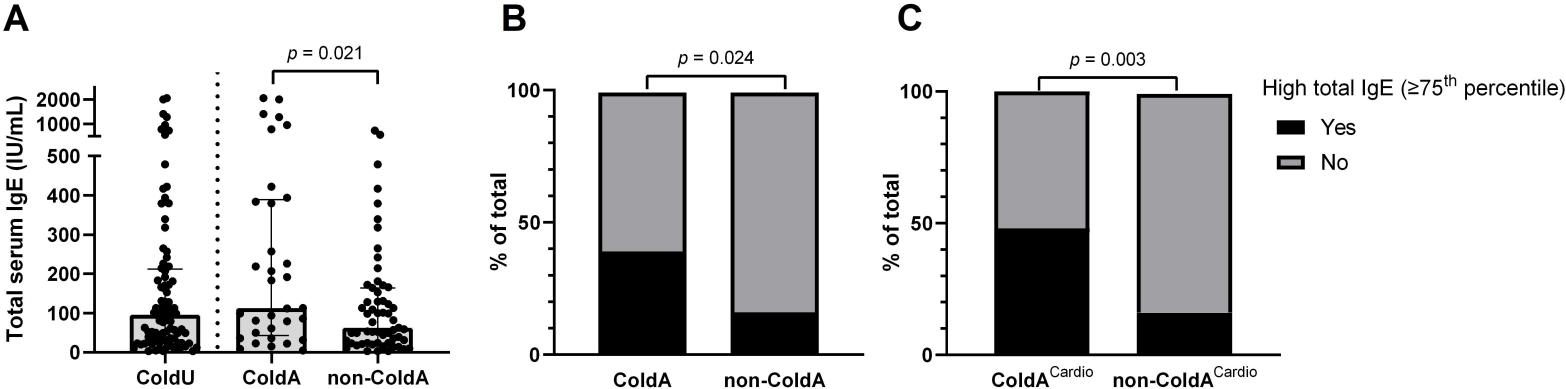
Total serum IgE levels and their association with ColdA. **(A)** Patients diagnosed with ColdA had significantly higher total serum IgE levels compared to those without (**Table 2**). **(B)** High total IgE levels, defined as being in the ≥75^th^ percentile (≥212.3 IU/mL), were associated with a higher frequency of ColdA (**Table 2**). **(C)** High total IgE levels were also associated with a higher frequency of ColdA with cardiac involvement. *ColdA*, cold-induced anaphylaxis; *ColdA^Cardio^*, cold-induced anaphylaxis with cardiac involvement; *ColdU*, cold urticaria; *IgE*, immunoglobulin E; *non-ColdA*, absence of cold-induced anaphylaxis.

Patients with ColdA had higher total serum IgE levels (median [IQR]: 113.0 [43.0−389.0] vs. 63.0 [24.0−164.0] IU/mL, *p* = 0.021, **Table 2**, **Figure 3A**) and a higher frequency of high total IgE, defined as ≥75^th^ percentile (39.4% [13/33] vs. 16.9% [10/59], *p* = 0.024; **Table 2**, **Figure 3B**), compared to non-ColdA patients. Patients with ColdA^Cardio^ also had higher total serum IgE levels (median [IQR]: 207.0 [33.5−408.0] vs. 79.0 [30.0−153.0] IU/mL, *p* = 0.022) and a higher frequency of high total IgE (48.0% [12/25] vs. 16.4% [11/67], *p* = 0.003, **Figure 3C**) compared to those without cardiac involvement.

### 3.8 Negative sCST was associated with lower ColdA frequency

ColdU with negative sCST (*n* = 39), compared to typical ColdU (*n* = 48), was associated with a lower frequency of ColdA (20.5% [8/39] vs. 52.1% [25/48], *p* = 0.004) and a higher frequency of spontaneous wheals (38.5% [15/39] vs. 4.2% [2/48], *p* < 0.001).

### 3.9 Negative sCST was not linked to features of autoinflammatory diseases

No significant differences were found between patients with negative sCST and those with typical ColdU in rates of recurrent fever or arthralgia (*n* = 0 for both groups), high CRP levels (≥5 mg/L; 10.3% [4/39] vs. 10.4% [5/48], *p* = 1.000), or CRP levels (median [IQR]: 0.90 [0.60−3.00] vs. 1.35 [0.73−3.48] mg/L, p = 0.225).

### 3.10 Sensitization to wasps or honeybees in ColdU patients

Elevated specific IgE levels to wasps (40.7%, 37/91) and honeybees (25.6%, 23/90) were detected among ColdU patients, with no significant differences in sensitization rates between ColdA and non-ColdA patients (**Table 2**). Among wasp- or honeybee-sensitized ColdU patients, 13% (6/46) experienced HVA, classified as Mueller grades I (*n* = 2), III (*n* = 1), and IV (*n* = 3) (**Supplementary Table S1**).

## 4 Discussion

To the best of our knowledge, this is the first study to systematically analyze BST levels, *KIT* p.D816V, HαT, and IgE levels in patients with ColdU and ColdA. Our findings provide new insights into the clinical and laboratory characteristics of these conditions.

Patients with typical ColdU were significantly more likely to experience ColdA than those with ColdU and negative sCST, a finding not previously reported. While negative sCST has been linked to hereditary systemic autoinflammatory diseases (49, 50), our study found no association with elevated CRP or other typical features of these disorders. Instead, negative sCST was associated with spontaneous wheals, suggesting that chronic spontaneous urticaria may be exacerbated by cold stimuli, a phenomenon not easily confirmed through sCST. ColdA patients exhibited a higher frequency of clinical features previously associated with ColdA in the COLD-CE study, including generalized wheals, skin angioedema, oropharyngeal/laryngeal manifestations, and itchy earlobes (2).

The *KIT* p.D816V variant, which is considered absent in the general population (51), was identified in 6.6% of ColdU patients and 6.3% of those with ColdA. Valent et al. highlighted that cold exposure might trigger anaphylaxis in individuals with SM, suggesting that temperature fluctuations can influence MC activity (52). Additionally, Akin et al. noted that hypotension during anaphylaxis raises suspicion of underlying clonal MC disease (36). Investigating clonal MC disease is standard practice in severe HVA cases (21, 53, 54). In our study, the presence of *KIT* p.D816V was not linked to increased ColdU severity, and thus it cannot currently be recommended as a routine test for assessing ColdA risk. Some patients with *KIT* p.D816V had normal BST levels, reinforcing the conclusion that normal BST levels do not exclude underlying clonal MC disease (39).

We observed a higher prevalence of HαT in ColdU and ColdA compared to the general population (24, 26, 32). Interestingly, the prevalence of HαT in ColdA exceeded that reported in patients with severe HVA (8.7%), where HαT has been linked to increased reaction severity (31). However, in our cohort, HαT was not associated with greater ColdU severity. The co-occurrence of HαT and *KIT* p.D816V in two out of six (33%) patients with *KIT* p.D816V is unlikely to be coincidental. Prior studies have reported this overlap (22, 31, 32, 43).

Previous research reported higher total IgE levels in patients with ColdU compared to those with chronic spontaneous urticaria (45). Our study found higher total IgE levels in ColdA patients compared to non-ColdA patients. Elevated IgE levels may reflect a type 2 immune response involving Th2 cells, eosinophils, MCs, and basophils, in which Th2 cytokines stimulate B cells to produce IgE (55, 56). However, in our study, atopic diseases, typically associated with Th2 dominance, were not linked to ColdA. While total IgE levels may serve as a potential biomarker for identifying ColdU patients at risk for severe reactions, the low specificity of total IgE testing limits its clinical utility (55).

The hypothesis that MC activation in ColdU is autoallergic and IgE-mediated, with cold triggering new autoallergens detected by IgE bound to MCs, remains compelling but unconfirmed. Supporting evidence includes the efficacy of omalizumab in ColdU (57) and earlier experiments showing that sensitivity to cold in some patients can be passively transferred, with particularly IgE suspected to play a role (14). Nevertheless, direct data validating this hypothesis are lacking.

The binding and cross-linking of the high-affinity receptor for IgE (**FcϵRI**) on MCs and basophils is crucial in many cases of anaphylaxis (10, 11, 58). Surface expression levels of FcϵRI on MCs are known to be positively regulated by IgE (45, 59), and significantly higher FcϵRI expression has been reported in patients with chronic inducible urticaria compared to controls (60). This further underscores the potential role of IgE in ColdA. Importantly, both IgE directed against exogenous antigens and autoreactive IgE antibodies have been found to elicit similar cellular responses (61), and IgE itself may enhance MC activation (62–65).

This study has several strengths, including the large cohort evaluated at a specialized academic center. However, limitations must be acknowledged. In patients with negative sCST, additional time-consuming tests with adjusted cold stimuli were not performed. Nonetheless, the diagnosis of ColdU was reliable based on consistent patient histories and documented photographs. Seasonal desensitization (66) may have led to falsely negative sCST results in patients exposed to cold air, and sCST was not repeated across different seasons. The diagnosis of ColdA relied on medical histories, as systematic generalized cold exposure testing would be unethical (1). Additionally, we lacked data on event-related tryptase levels (67), as such evaluations are rarely conducted systematically in this patient population (1). Variability in IgE levels due to demographic factors such as age and gender (45, 68) was also not assessed.

Assessing BST levels may help identify patients with HαT and, to a lesser extent, the *KIT* p.D816V variant. Our findings demonstrate a higher prevalence of HαT and *KIT* p.D816V in ColdU and ColdA compared to the general population. These results raise the hypothesis that HαT and clonal MC disease may contribute to the pathogenesis of ColdU and ColdA in some patients. Additionally, elevated IgE levels could serve as a potential biomarker for ColdA. Further research is needed to clarify the clinical significance of these genetic and immunological findings.

## Data Availability

The datasets presented in this article are not readily available because of privacy or ethical restrictions. Requests to access the datasets should be directed to MB, mojca.bizjak@klinika-golnik.si.

## References

[1] Bizjak M Rutkowski K Asero R. Risk of anaphylaxis associated with cold urticaria. Curr Treat Options Allergy. (2024) 11:167–75. doi: 10.1007/s40521-024-00366-9

[2] Bizjak M Kosnik M Dinevski D Thomsen SF Fomina D Borzova E. Risk factors for systemic reactions in typical cold urticaria: Results from the COLD-CE study. Allergy. (2022) 77:2185–99. doi: 10.1111/all.15194 34862605

[3] Prosty C Gabrielli S Le M Ensina LF Zhang X Netchiporouk E. Prevalence, management, and anaphylaxis risk of cold urticaria: A systematic review and meta-analysis. J Allergy Clin Immunol Pract. (2022) 10:586–96 e4. doi: 10.1016/j.jaip.2021.10.012 34673287

[4] Bizjak M Kosnik M Dinevski D Thomsen SF Fomina D Borzova E. Adrenaline autoinjector is underprescribed in typical cold urticaria patients. Allergy. (2022) 77:2224–9. doi: 10.1111/all.15274 35258111

[5] Maltseva N Borzova E Fomina D Bizjak M Terhorst-Molawi D Kosnik M. Cold urticaria - What we know and what we do not know. Allergy. (2021) 76:1077–94. doi: 10.1111/all.14674 33249577

[6] Magerl M Altrichter S Borzova E Gimenez-Arnau A Grattan CE Lawlor F. The definition, diagnostic testing, and management of chronic inducible urticarias - The EAACI/GA(2) LEN/EDF/UNEV consensus recommendations 2016 update and revision. Allergy. (2016) 71(6):780–802. doi: 10.1111/all.12884 26991006

[7] Relvas M Silva J Alves F Matos A Bizjak M Goncalo M. Localized cold urticaria: an unusual form of cold urticaria. J Eur Acad Dermatol Venereol. (2022) 36:e133–e5. doi: 10.1111/jdv.17697 34551454

[8] Benelli E Longo G Barbi E Berti I. Anaphylaxis in atypical cold urticaria: case report and review of literature. Ital J Pediatr. (2018) 44:135. doi: 10.1186/s13052-018-0578-6 30424814 PMC6234673

[9] Golden DBK Wang J Waserman S Akin C Campbell RL Ellis AK. Anaphylaxis: A 2023 practice parameter update. Ann Allergy Asthma Immunol. (2024) 132:124–76. doi: 10.1016/j.anai.2023.09.015 38108678

[10] Galli SJ Tsai M. IgE and mast cells in allergic disease. Nat Med. (2012) 18:693–704. doi: 10.1038/nm.2755 22561833 PMC3597223

[11] Olivera A Beaven MA Metcalfe DD. Mast cells signal their importance in health and disease. J Allergy Clin Immunol. (2018) 142:381–93. doi: 10.1016/j.jaci.2018.01.034 29454835

[12] Soter NA Wasserman SI Austen KF. Cold urticaria: release into the circulation of histamine and eosinophil chemotactic factor of anaphylaxis during cold challenge. N Engl J Med. (1976) 294:687–90. doi: 10.1056/NEJM197603252941302 55969

[13] Kaplan AP Gray L Shaff RE Horakova Z Beaven MA. *In vivo* studies of mediator release in cold urticaria and cholinergic urticaria. J Allergy Clin Immunol. (1975) 55:394–402. doi: 10.1016/0091-6749(75)90078-0 48522

[14] Kaplan AP Garofalo J Sigler R Hauber T. Idiopathic cold urticaria: *in vitro* demonstration of histamine release upon challenge of skin biopsies. N Engl J Med. (1981) 305:1074–7. doi: 10.1056/NEJM198110293051808 6168912

[15] Bentley-Phillips CB Black AK Greaves MW. Induced tolerance in cold urticaria caused by cold-evoked histamine release. Lancet. (1976) 2:63–6. doi: 10.1016/S0140-6736(76)92285-6 59149

[16] Kaplan AP Beaven MA. *In vivo* studies of the pathogenesis of cold urticaria, cholinergic urticaria, and vibration-induced swelling. J Invest Dermatol. (1976) 67:327–32. doi: 10.1111/1523-1747.ep12514352 61247

[17] Heavey DJ Kobza-Black A Barrow SE Chappell CG Greaves MW Dollery CT. Prostaglandin D2 and histamine release in cold urticaria. J Allergy Clin Immunol. (1986) 78:458–61. doi: 10.1016/0091-6749(86)90033-3 2428856

[18] Kaplan AP. The pathogenic basis of urticaria and angioedema: recent advances. Am J Med. (1981) 70:755–8. doi: 10.1016/0002-9343(81)90528-3 7211911

[19] Gonzalez-de-Olano D Navarro-Navarro P Munoz-Gonzalez JI Sanchez-Munoz L Henriques A de-Andres-Martin A. Clinical impact of the TPSAB1 genotype in mast cell diseases: A REMA study in a cohort of 959 individuals. Allergy. (2024) 79:711–23. doi: 10.1111/all.15911 37818990

[20] Lyons JJ. Hereditary alpha tryptasemia: genotyping and associated clinical features. Immunol Allergy Clin North Am. (2018) 38:483–95. doi: 10.1016/j.iac.2018.04.003 PMC6411063 30007465

[21] Kačar M Rijavec M Šelb J Korošec P. Clonal mast cell disorders and hereditary alpha-tryptasemia as risk factors for anaphylaxis. Clin Exp Allergy. (2023) 53(4):392–404. doi: 10.1111/cea.14264 36654513

[22] Lyons JJ Chovanec J O’Connell MP Liu Y Selb J Zanotti R. Heritable risk for severe anaphylaxis associated with increased alpha-tryptase-encoding germline copy number at TPSAB1. J Allergy Clin Immunol. (2021) 147:622–32. doi: 10.1016/j.jaci.2020.06.035 32717252

[23] Chovanec J Tunc I Hughes J Halstead J Mateja A Liu Y. Genetically determining individualized clinical reference ranges for the biomarker tryptase can limit unnecessary procedures and unmask myeloid neoplasms. Blood Adv. (2023) 7(9):1796–810. doi: 10.1182/bloodadvances.2022007936 PMC10164828 36170795

[24] Valent P Hoermann G Bonadonna P Hartmann K Sperr WR Broesby-Olsen S. The normal range of baseline tryptase should be 1-15 ng/ml and covers healthy individuals with hereditary alpha tryptasemia. J Allergy Clin Immunol Pract. (2023) 11(10):3010–20. doi: 10.1016/j.jaip.2023.08.008 PMC11869997 37572755

[25] Lyons JJ Yu X Hughes JD Le QT Jamil A Bai Y. Elevated basal serum tryptase identifies a multisystem disorder associated with increased TPSAB1 copy number. Nat Genet. (2016) 48:1564–9. doi: 10.1038/ng.3696 PMC5397297 27749843

[26] Lyons JJ Greiner G Hoermann G Metcalfe DD. Incorporating tryptase genotyping into the workup and diagnosis of mast cell diseases and reactions. J Allergy Clin Immunol Pract. (2022) 10:1964–73. doi: 10.1016/j.jaip.2022.05.003 PMC11837873 35597543

[27] Le QT Lyons JJ Naranjo AN Olivera A Lazarus RA Metcalfe DD. Impact of naturally forming human alpha/beta-tryptase heterotetramers in the pathogenesis of hereditary alpha-tryptasemia. J Exp Med. (2019) 216:2348–61. doi: 10.1084/jem.20190701 PMC6780998 31337736

[28] Glover SC Carter MC Korosec P Bonadonna P Schwartz LB Milner JD. Clinical relevance of inherited genetic differences in human tryptases: Hereditary alpha-tryptasemia and beyond. Ann Allergy Asthma Immunol. (2021) 127:638–47. doi: 10.1016/j.anai.2021.08.009 PMC9413800 34400315

[29] Arock M Hoermann G Sotlar K Hermine O Sperr WR Hartmann K. Clinical impact and proposed application of molecular markers, genetic variants, and cytogenetic analysis in mast cell neoplasms: Status 2022. J Allergy Clin Immunol. (2022) 149:1855–65. doi: 10.1016/j.jaci.2022.04.004 35430191

[30] Sabato V Chovanec J Faber M Milner JD Ebo D Lyons JJ. First identification of an inherited TPSAB1 quintuplication in a patient with clonal mast cell disease. J Clin Immunol. (2018) 38:457–9. doi: 10.1007/s10875-018-0506-y 29748908

[31] Korošec P Sturm GJ Lyons JJ Marolt TP Svetina M Košnik M. High burden of clonal mast cell disorders and hereditary alpha-tryptasemia in patients who need Hymenoptera venom immunotherapy. Allergy. (2024) 79(9):2458–69. doi: 10.1111/all.16084 PMC11939115 38477502

[32] Chollet MB Akin C. Hereditary alpha tryptasemia is not associated with specific clinical phenotypes. J Allergy Clin Immunol. (2022) 149:728–35 e2. doi: 10.1016/j.jaci.2021.06.017 34174297

[33] Valent P Hartmann K Schwaab J Alvarez-Twose I Brockow K Bonadonna P. Personalized management strategies in mast cell disorders: ECNM-AIM user’s guide for daily clinical practice. J Allergy Clin Immunol Pract. (2022) 10:1999–2012 e6. doi: 10.1016/j.jaip.2022.03.007 35342031

[34] Valent P Akin C Metcalfe DD. Mastocytosis: 2016 updated WHO classification and novel emerging treatment concepts. Blood. (2017) 129:1420–7. doi: 10.1182/blood-2016-09-731893 PMC5356454 28031180

[35] Valent P Akin C Hartmann K Alvarez-Twose I Brockow K Hermine O. Updated diagnostic criteria and classification of mast cell disorders: A consensus proposal. Hemisphere. (2021) 5:e646. doi: 10.1097/HS9.0000000000000646 PMC8659997 34901755

[36] Akin C. Mast cell activation syndromes. J Allergy Clin Immunol. (2017) 140:349–55. doi: 10.1016/j.jaci.2017.06.007 28780942

[37] Gulen T Akin C. Anaphylaxis and mast cell disorders. Immunol Allergy Clin North Am. (2022) 42:45–63. doi: 10.1016/j.iac.2021.09.007 34823750

[38] Volertas S CFt S Akin C. New insights into clonal mast cell disorders including mastocytosis. Immunol Allergy Clin North Am. (2018) 38:341–50. doi: 10.1016/j.iac.2018.04.014 30007455

[39] Selb J Rijavec M Erzen R Zidarn M Kopac P Skerget M. Routine KIT p.D816V screening identifies clonal mast cell disease in patients with Hymenoptera allergy regularly missed using baseline tryptase levels alone. J Allergy Clin Immunol. (2021) 148:621–6 e7. doi: 10.1016/j.jaci.2021.02.043 33753098 PMC10964493

[40] Hoermann G Sotlar K Jawhar M Kristensen T Bachelot G Nedoszytko B. Standards of genetic testing in the diagnosis and prognostication of systemic mastocytosis in 2022: recommendations of the EU-US cooperative group. J Allergy Clin Immunol Pract. (2022) 10:1953–63. doi: 10.1016/j.jaip.2022.03.001 35283331

[41] Arock M Sotlar K Akin C Broesby-Olsen S Hoermann G Escribano L. KIT mutation analysis in mast cell neoplasms: recommendations of the European Competence Network on Mastocytosis. Leukemia. (2015) 29:1223–32. doi: 10.1038/leu.2015.24 PMC4522520 25650093

[42] Navarro-Navarro P Álvarez-Twose I Pérez-Pons A Henriques A Mayado A García-Montero AC. KITD816V mutation in blood for the diagnostic screening of systemic mastocytosis and mast cell activation syndromes. Allergy. (2023) 78(5):1347–59. doi: 10.1111/all.15584 36385619

[43] Sturm GJ SChadelbauer E Marta G Bonadonna P Kosnik M. Risk factors for severe sting reactions and side effects during venom immunotherapy. J Allergy Clin Immunol Pract. (2025) 13(1):17-23. doi: 10.1016/j.jaip.2024.08.025 39173970

[44] Bizjak M Maurer M Kosnik M Terhorst-Molawi D Zver S Burmeister T. Severe cold urticaria can point to an underlying clonal mast cell disorder. Allergy. (2021) 76:2609–13. doi: 10.1111/all.14844 33797762

[45] Bizjak M Kosnik M. Key differences between chronic inducible and spontaneous urticaria. Front Allergy. (2024) 5:1487831. doi: 10.3389/falgy.2024.1487831 39483682 PMC11524999

[46] Kristensen T Vestergaard H Moller MB. Improved detection of the KIT D816V mutation in patients with systemic mastocytosis using a quantitative and highly sensitive real-time qPCR assay. J Mol Diagn. (2011) 13:180–8. doi: 10.1016/j.jmoldx.2010.10.004 PMC3279709 21354053

[47] Svetina M Selb J Lyons JJ Korosec P Rijavec M. Clinically accessible amplitude-based multiplex ddPCR assay for tryptase genotyping. Sci Rep. (2024) 14:2416. doi: 10.1038/s41598-024-52983-8 38287122 PMC10825142

[48] Valent P Escribano L Broesby-Olsen S Hartmann K Grattan C Brockow K. Proposed diagnostic algorithm for patients with suspected mastocytosis: a proposal of the European Competence Network on Mastocytosis. Allergy. (2014) 69:1267–74. doi: 10.1111/all.2014.69.issue-10 24836395

[49] Diaz VL Gribbons KB Yazdi-Nejad K Kuemmerle-Deschner J Wanderer AA Broderick L. Cold urticaria syndromes: diagnosis and management. J Allergy Clin Immunol Pract. (2023) 11:2275–85. doi: 10.1016/j.jaip.2023.05.040 37290539

[50] Kacar M Pathak S Savic S. Hereditary systemic autoinflammatory diseases and Schnitzler’s syndrome. Rheumatol (Oxford). (2019) 58:vi31–43. doi: 10.1093/rheumatology/kez448 PMC6878846 31769858

[51] Kristensen T Vestergaard H Bindslev-Jensen C Moller MB Broesby-Olsen S Mastocytosis Centre OUH. Sensitive KIT D816V mutation analysis of blood as a diagnostic test in mastocytosis. Am J Hematol. (2014) 89:493–8. doi: 10.1002/ajh.23672 24443360

[52] Valent P. Risk factors and management of severe life-threatening anaphylaxis in patients with clonal mast cell disorders. Clin Exp Allergy. (2014) 44:914–20. doi: 10.1111/cea.2014.44.issue-7 PMC4603355 24702655

[53] Stoevesandt J Sturm GJ Bonadonna P Oude Elberink JNG Trautmann A. Risk factors and indicators of severe systemic insect sting reactions. Allergy. (2020) 75:535–45. doi: 10.1111/all.13945 31194889

[54] Kosnik M Korosec P. Venom immunotherapy: clinical efficacy, safety and contraindications. Expert Rev Clin Immunol. (2015) 11:877–84. doi: 10.1586/1744666X.2015.1052409 26018865

[55] Guida G Bagnasco D Carriero V Bertolini F Ricciardolo FLM Nicola S. Critical evaluation of asthma biomarkers in clinical practice. Front Med (Lausanne). (2022) 9:969243. doi: 10.3389/fmed.2022.969243 36300189 PMC9588982

[56] Jutel M Agache I Zemelka-Wiacek M Akdis M Chivato T Del Giacco S. Nomenclature of allergic diseases and hypersensitivity reactions: Adapted to modern needs: An EAACI position paper. Allergy. (2023) 78:2851–74. doi: 10.1111/all.v78.11 37814905

[57] Kulthanan K Hunnangkul S Tuchinda P Chularojanamontri L Weerasubpong P Subchookul C. Treatments of cold urticaria: A systematic review. J Allergy Clin Immunol. (2019) 143:1311–31. doi: 10.1016/j.jaci.2019.02.005 30776418

[58] Shaker MS Wallace DV Golden DBK Oppenheimer J Bernstein JA Campbell RL. Anaphylaxis-a 2020 practice parameter update, systematic review, and Grading of Recommendations, Assessment, Development and Evaluation (GRADE) analysis. J Allergy Clin Immunol. (2020) 145:1082–123. doi: 10.1016/j.jaci.2020.01.017 32001253

[59] Tanaka S Furuta K. Roles of igE and histamine in mast cell maturation. Cells. (2021) 10(8):2170. doi: 10.3390/cells10082170 34440939 PMC8392195

[60] Giménez-Arnau AM Ribas-Llauradó C Mohammad-Porras N Deza G Pujol RM Gimeno R. IgE and high-affinity IgE receptor in chronic inducible urticaria, pathogenic, and management relevance. Clin Transl Allergy. (2022) 12(2):e12117. doi: 10.1002/clt2.12117 35126995 PMC8805593

[61] Maurer M Altrichter S Schmetzer O Scheffel J Church MK Metz M. Immunoglobulin E-mediated autoimmunity. Front Immunol. (2018) 9:689. doi: 10.3389/fimmu.2018.00689 29686678 PMC5900004

[62] Akin C Scott LM Kocabas CN Kushnir-Sukhov N Brittain E Noel P. Demonstration of an aberrant mast-cell population with clonal markers in a subset of patients with “idiopathic” anaphylaxis. Blood. (2007) 110:2331–3. doi: 10.1182/blood-2006-06-028100 PMC1988935 17638853

[63] Kalesnikoff J Huber M Lam V Damen JE Zhang J Siraganian RP. Monomeric IgE stimulates signaling pathways in mast cells that lead to cytokine production and cell survival. Immunity. (2001) 14:801–11. doi: 10.1016/S1074-7613(01)00159-5 11420049

[64] Lam V Kalesnikoff J Lee CW Hernandez-Hansen V Wilson BS Oliver JM. IgE alone stimulates mast cell adhesion to fibronectin via pathways similar to those used by IgE + antigen but distinct from those used by Steel factor. Blood. (2003) 102:1405–13. doi: 10.1182/blood-2002-10-3176 12702510

[65] Pandey V Mihara S Fensome-Green A Bolsover S Cockcroft S. Monomeric IgE stimulates NFAT translocation into the nucleus, a rise in cytosol Ca2+, degranulation, and membrane ruffling in the cultured rat basophilic leukemia-2H3 mast cell line. J Immunol. (2004) 172:4048–58. doi: 10.4049/jimmunol.172.7.4048 15034016

[66] Kring Tannert L Stahl Skov P Bjerremann Jensen L Maurer M Bindslev-Jensen C. Cold urticaria patients exhibit normal skin levels of functional mast cells and histamine after tolerance induction. Dermatology. (2012) 224:101–5. doi: 10.1159/000336572 22398751

[67] Valent P Akin C Bonadonna P Hartmann K Brockow K Niedoszytko M. Proposed diagnostic algorithm for patients with suspected mast cell activation syndrome. J Allergy Clin Immunol Pract. (2019) 7:1125–33 e1. doi: 10.1016/j.jaip.2019.01.006 30737190 PMC6643056

[68] Altrichter S Fok JS Jiao Q Kolkhir P Pyatilova P Romero SM. Total igE as a marker for chronic spontaneous urticaria. Allergy Asthma Immunol Res. (2021) 13:206–18. doi: 10.4168/aair.2021.13.2.206 PMC7840871 33474856

