# Supplementary material for: Cold-induced anaphylaxis: new insights into clinical and genetic characteristics
Source: Front Immunol. 2025 Feb 21;16:1558284. doi: 10.3389/fimmu.2025.1558284 (PMC11885499; doi:10.3389/fimmu.2025.1558284)
Supplement: Supplementary file 1 [file Table1.docx]

**Supplementary Table S1. Clinical and laboratory characteristics of individual ColdU patients.**

| Nr. | Age (y) | Gender | Typical ColdU (*n* = 48) | ColdA (*n* = 33) | ColdA^Cardio^ (*n* = 25) | Systemic reaction to *Hymenoptera* venom (*n* = 6)^a^ | Atopic disease (*n* = 30) | Total serum IgE (IU/mL) | sIgE honeybee, i1 (IU/mL) | sIgE wasp, i3 (IU/mL) | BST l(ng/mL) | *KIT* p.D816V (*n* = 6) | HαT *(n* = 10)^b^ | Genotype encoded at  *TPSAB1* | Genotype encoded at  *TPSAB1* and *TPSB2* |
| --- | --- | --- | --- | --- | --- | --- | --- | --- | --- | --- | --- | --- | --- | --- | --- |
| 1 | 43 | f | **Y** | **Y** | **Y** | N | Y | 183 | <0.10 | 0.683 | 3.66 | N | *^Nd^* | *^Nd^* | *^Nd^* |
| 2 | 38 | m | **Y** | **Y** | **Y** | N | Y | 2050 | 1.94 | 0.188 | 3.24 | N | *^Nd^* | *^Nd^* | *^Nd^* |
| 3 | 18 | f | **Y** | **Y** | **Y** | N | Y | 953 | <0.10 | <0.10 | 3.41 | N | *^Nd^* | *^Nd^* | *^Nd^* |
| 4 | **24** | **m** | **Y** | **Y** | **Y** | **N** | **Y** | **1282** | **0.339** | **0.142** | **11.60** | **N** | **Y** | **αα/α** | **αα,β/α,β** |
| 5 | 73 | f | **Y** | **Y** | **Y** | N | N | 207 | <0.10 | 0.2 | 8.15 | N | N | α/β | α,β/β,β |
| 6 | **61** | **f** | **Y** | **Y** | **Y** | **N** | **Y** | **9** | **<0.10** | **<0.10** | **15.90** | N | **Y** | **αα/α** | **αα,β/α,β** |
| 7 | 51 | m | **Y** | **Y** | **Y** | **Y(I)** | N | 257 | *^Nd^* | 2.29 | 5.34 | N | *^Nd^* | *^Nd^* | *^Nd^* |
| 8 | 23 | f | **Y** | **Y** | **Y** | N | Y | 94 | <0.10 | <0.10 | 5.32 | N | *^Nd^* | *^Nd^* | *^Nd^* |
| 9 | 47 | f | N | **Y** | **Y** | N | N | 1407 | <0.10 | 0.145 | 7.57 | N | N | α/α | α,β/α,β |
| 10 | 43 | m | **Y** | **Y** | N | N | N | 81 | <0.10 | 1.12 | 6.19 | N | N | β/β | β,β/β,β |
| 11 | **65** | **f** | **Y** | **Y** | **N** | **N** | **N** | **113** | **<0.10** | **<0.10** | **23.00** | **Y** | **N** | **α/β** | **α,β/β,β** |
| 12 | 49 | f | **Y** | N | N | N | N | 164 | <0.10 | 6.2 | 3.57 | N | *^Nd^* | *^Nd^* | *^Nd^* |
| 13 | **35** | **m** | **N** | **N** | **N** | **N** | **N** | **52** | **<0.10** | **<0.10** | **8.53** | **Y** | **N** | **α/α** | **α,β/α,β** |
| 14 | 46 | f | N | N | N | N | Y | 52 | 1.58 | 4.2 | 5.35 | N | *^Nd^* | *^Nd^* | *^Nd^* |
| 15 | 27 | f | N | N | N | N | N | 12 | <0.10 | <0.10 | 3.83 | N | *^Nd^* | *^Nd^* | *^Nd^* |
| 16 | 40 | m | **Y** | N | N | N | Y | 128 | 0.736 | 0.437 | 5.50 | N | *^Nd^* | *^Nd^* | *^Nd^* |
| 17 | 40 | m | N | N | N | N | N | 123 | <0.10 | 0.468 | 7.79 | N | N | α/β | α,β/β,β |
| 18 | 58 | m | **Y** | N | N | N | N | 82 | 3.51 | 24.1 | 4.44 | N | *^Nd^* | *^Nd^* | *^Nd^* |
| 19 | **56** | **f** | **N** | **N** | **N** | **N** | **N** | **172** | **<0.10** | **1.510** | **13.60** | **N** | **Y** | **αα/α** | **αα,β/α,β** |
| 20 | 42 | f | N | N | N | N | Y | 559 | <0.10 | <0.10 | 5.49 | N | *^Nd^* | *^Nd^* | *^Nd^* |
| 21 | 43 | f | N | **Y** | N | N | N | 61 | *^Nd^* | *^Nd^* | 3.96 | N | *^Nd^* | *^Nd^* | *^Nd^* |
| 22 | 52 | f | **Y** | N | N | N | N | 24 | <0.10 | <0.10 | 6.37 | N | N | α/β | α,β/β,β |
| 23 | 56 | f | N | N | N | N | Y | 49 | 2.62 | 0.146 | 5.55 | N | *^Nd^* | *^Nd^* | *^Nd^* |
| 24 | 41 | f | **Y** | N | N | N | Y | 24 | 1.54 | 3.110 | 7.41 | N | N | α/α | α,β/α,β |
| 25 | 57 | f | **Y** | N | N | N | N | 21 | 1.22 | 0.723 | 6.00 | N | N | α/β | α,β/β,β |
| 26 | 40 | f | N | N | N | N | N | 18 | <0.10 | <0.10 | 4.55 | N | *^Nd^* | *^Nd^* |  |
| 27 | **47** | **m** | **N** | **N** | **N** | **N** | **N** | **166** | **<0.10** | **1.270** | **12.7** | **N** | **Y** | **αα/β** | **αα,β/β,β** |
| 28 | 26 | f | **Y** | N | N | N | Y | 113 | <0.10 | <0.10 | 6.09 | N | N | α/β | α,β/β,β |
| 29 | 38 | f | N | N | N | N | N | 181 | <0.10 | <0.10 | 3.05 | N | *^Nd^* | *^Nd^* | *^Nd^* |
| 30 | 26 | m | **Y** | N | N | N | Y | 44 | <0.10 | <0.10 | 2.92 | N | *^Nd^* | *^Nd^* | *^Nd^* |
| 31 | 33 | f | **Y** | **Y** | N | N | N | 36 | <0.10 | 1.630 | 5.10 | N | *^Nd^* | *^Nd^* | *^Nd^* |
| 32 | 30 | m | N | N | N | N | N | 31 | 0.358 | 0.719 | 2.90 | N | *^Nd^* | *^Nd^* | *^Nd^* |
| 33 | 22 | m | **Y** | N | N | N | N | 99 | <0.10 | 1.620 | 6.06 | N | N | α/β | α,β/β,β |
| 34 | 30 | f | **Y** | N | N | N | N | 14 | <0.10 | <0.10 | 5.33 | N | *^Nd^* | *^Nd^* | *^Nd^* |
| 35 | **28** | **f** | **Y** | **Y** | **N** | **N** | **Y** | **2000** | **1.9** | **<0.10** | **14.00** | **N** | **Y** | **αα/α** | **αα,β/α,β** |
| 36 | 21 | f | N | N | N | N | N | 23 | <0.10 | <0.10 | 3.87 | N | *^Nd^* | *^Nd^* | *^Nd^* |
| 37 | 18 | f | N | N | N | N | Y | 379 | <0.10 | 1.29 | 6.59 | N | N | α/β | α,β/β,β |
| 38 | 23 | f | N | **Y** | **Y** | N | N | 31 | <0.10 | 1.23 | 4.72 | *^Nd^* | *^Nd^* | *^Nd^* | *^Nd^* |
| 39 | 45 | f | **Y** | **Y** | **Y** | N | N | 23 | 0.52 | 0.54 | 5.30 | N | *^Nd^* | *^Nd^* | *^Nd^* |
| 40 | 24 | m | N | N | N | N | N | 59 | 12.0 | 0.262 | 5.81 | N | *^Nd^* | *^Nd^* | *^Nd^* |
| 41 | 53 | f | N | N | N | N | N | 3 | <0.10 | <0.10 | 3.73 | N | *^Nd^* | *^Nd^* | *^Nd^* |
| 42 | 59 | m | **Y** | **Y** | **Y** | **Y (IV)** | N | 192 | 1.960 | 1.4 | 3.37 | N | *^Nd^* | *^Nd^* | *^Nd^* |
| 43 | 28 | m | **Y** | N | N | N | N | 100 | <0.10 | <0.10 | 3.20 | N | *^Nd^* | *^Nd^* | *^Nd^* |
| 44 | 37 | m | N | N | N | N | N | 8 | <0.10 | <0.10 | 2.66 | N | *^Nd^* | *^Nd^* | *^Nd^* |
| 45 | 18 | m | N | N | N | N | N | 13 | <0.10 | <0.10 | 2.59 | N | *^Nd^* | *^Nd^* | *^Nd^* |

**Supplementary Table S1.** *Cont.*

| Nr. | Age (y) | Gender | Typical ColdU (*n* = 48) | ColdA (*n* = 33) | ColdA^Cardio^ (*n* = 25) | Systemic reaction to *Hymenoptera* venom (*n* = 6)^a^ | Atopic disease (*n* = 30) | Total serum IgE (IU/mL) | sIgE honeybee, i1 (IU/mL) | sIgE wasp, i3 (IU/mL) | BST (ng/mL) | *KIT* p.D816V (*n* = 6) | HαT *(n* = 10)^b^ | Genotype encoded at  *TPSAB1* | Genotype encoded at  *TPSAB1* and *TPSB2* |
| --- | --- | --- | --- | --- | --- | --- | --- | --- | --- | --- | --- | --- | --- | --- | --- |
| 46 | **47** | **f** | **N** | **N** | **N** | **Y (IV)** | **Y** | **24** | **0.960** | **5.620** | **27.80** | **Y** | **Y** | **αα/β** | **αα,β/β,β** |
| 47 | 41 | f | **Y** | **Y** | **Y** | N | Y | 36 | <0.10 | <0.10 | 3.42 | N | *^Nd^* | *^Nd^* | *^Nd^* |
| 48 | 31 | f | **Y** | **Y** | **Y** | N | N | 15 | <0.10 | 0.351 | 1.19 | N | *^Nd^* | *^Nd^* | *^Nd^* |
| 49 | 72 | m | N | N | N | N | N | 77 | 0.166 | <0.10 | 5.57 | N | *^Nd^* | *^Nd^* | *^Nd^* |
| 50 | 32 | f | **Y** | **Y** | **Y** | N | N | 226 | 0.182 | 0.936 | 2.28 | N | *^Nd^* | *^Nd^* | *^Nd^* |
| 51 | 26 | f | N | N | N | N | Y | 49 | 0.806 | <0.10 | 1.88 | N | *^Nd^* | *^Nd^* | *^Nd^* |
| 52 | 53 | m | N | **Y** | **Y** | **Y (III)** | N | 422 | 8.07 | 6.30 | 3.63 | N | *^Nd^* | *^Nd^* | *^Nd^* |
| 53 | 39 | f | **Y** | **Y** | **Y** | N | N | 22 | <0.10 | <0.10 | 4.67 | N | *^Nd^* | *^Nd^* | *^Nd^* |
| 54 | 21 | f | N | N | N | N | Y | 171 | <0.10 | <0.10 | 3.24 | N | *^Nd^* | *^Nd^* | *^Nd^* |
| 55 | 55 | f | **Y** | **Y** | **Y** | N | N | 394 | 0.168 | <0.10 | 5.70 | N | *^Nd^* | *^Nd^* | *^Nd^* |
| 56 | 35 | f | N | N | N | N | N | 10 | <0.10 | <0.10 | 4.09 | N | *^Nd^* | *^Nd^* | *^Nd^* |
| 57 | 54 | f | N | N | N | N | N | 10 | <0.10 | 0.521 | 4.63 | N | *^Nd^* | *^Nd^* | *^Nd^* |
| 58 | 31 | f | N | N | N | N | N | 109 | 0.127 | 5.79 | 1.74 | N | *^Nd^* | *^Nd^* | *^Nd^* |
| 59 | 42 | f | N | **Y** | N | N | N | 112 | <0.10 | <0.10 | 3.64 | N | *^Nd^* | *^Nd^* | *^Nd^* |
| 60 | 33 | f | N | N | N | N | N | 101 | <0.10 | 0.927 | 3.84 | N | *^Nd^* | *^Nd^* | *^Nd^* |
| 61 | 23 | f | N | N | N | N | N | 20 | <0.10 | 1.89 | 2.17 | N | *^Nd^* | *^Nd^* | *^Nd^* |
| 62 | 29 | f | **Y** | **Y** | **Y** | N | N | 5 | <0.10 | <0.10 | 4.41 | N | *^Nd^* | *^Nd^* | *^Nd^* |
| 63 | 26 | f | **Y** | N | N | **Y (IV)** | N | 39 | <0.10 | 29.4 | 4.65 | N | *^Nd^* | *^Nd^* | *^Nd^* |
| 64 | 48 | f | **Y** | N | N | N | N | 318 | <0.10 | <0.10 | 10.30 | N | N | α/β | α,β/β,β |
| 65 | 38 | f | **Y** | **Y** | **Y** | N | N | 380 | <0.10 | <0.10 | 4.16 | N | *^Nd^* | *^Nd^* | *^Nd^* |
| 66 | **62** | **f** | **Y** | **Y** | **N** | **N** | **N** | **86** | **<0.10** | **25.6** | **17.10** | **N** | **Y** | **αα/α** | **αα,β/α,β** |
| 67 | 50 | f | N | N | N | N | N | 3 | <0.10 | <0.10 | 5.91 | N | *^Nd^* | *^Nd^* | *^Nd^* |
| 68 | 34 | f | N | **Y** | N | N | N | 79 | <0.10 | <0.10 | 4.43 | N | *^Nd^* | *^Nd^* | *^Nd^* |
| 69 | 43 | m | **Y** | **Y** | **Y** | N | N | 219 | 4.16 | 2.10 | 5.02 | N | *^Nd^* | *^Nd^* | *^Nd^* |
| 70 | 26 | m | N | N | N | N | N | 53 | <0.10 | <0.10 | 1.92 | N | *^Nd^* | α/β | α,β/β,β |
| 71 | 38 | f | **Y** | N | N | N | Y | 63 | <0.10 | <0.10 | 1.59 | N | *^Nd^* | *^Nd^* | *^Nd^* |
| 72 | **42** | **f** | **Y** | **N** | **N** | **N** | **Y** | **3** | **<0.10** | **<0.10** | **11.10** | **N** | **Y** | **αα/α** | **αα,β/α,β** |
| 73 | 30 | m | **Y** | N | N | N | Y | 726 | 0.686 | 0.596 | 5.61 | N | *^Nd^* | *^Nd^* | *^Nd^* |
| 74 | 40 | m | N | N | N | N | Y | 153 | <0.10 | <0.10 | 2.99 | N | *^Nd^* | *^Nd^* | *^Nd^* |
| 75 | 27 | f | **Y** | N | N | N | Y | 242 | 3.14 | 0.454 | 3.23 | N | *^Nd^* | *^Nd^* | *^Nd^* |
| 76 | **58** | **f** | **N** | **Y** | **Y** | **N** | **Y** | **50** | **<0.10** | **<0.10** | **13.20** | **Y** | **Y** | **αα/β** | **αα,β/β,β** |
| 77 | 58 | f | **Y** | **Y** | **Y** | N | Y | 384 | 0.141 | <0.10 | 6.02 | N | N | β/β | *^Nd^* |
| 78 | 30 | m | **Y** | **Y** | **Y** | N | Y | 99 | <0.10 | 0.671 | 2.08 | N | *^Nd^* | *^Nd^* | *^Nd^* |
| 79 | 66 | m | N | N | N | N | N | 113 | <0.10 | <0.10 | 7.63 | N | N | β/β | β,β/β,β |
| 80 | **35** | **f** | **Y** | **N** | **N** | **N** | **N** | **36** | **<0.10** | **3.7** | **10.8** | **N** | **Y** | **αα/α** | **αα,β/α,β** |
| 81 | 31 | f | N | N | N | N | N | 57 | <0.10 | <0.10 | 4.33 | N | *^Nd^* | *^Nd^* | *^Nd^* |
| 82 | 41 | f | **Y** | N | N | N | N | 98 | <0.10 | 33.6 | 3.16 | N | *^Nd^* | *^Nd^* | *^Nd^* |
| 83 | 64 | f | **Y** | N | N | N | N | 214 | 4.62 | 52.2 | 2.91 | N | *^Nd^* | *^Nd^* | *^Nd^* |
| 84 | **56** | **m** | **Y** | **N** | **N** | **Y (I)** | **Y** | **479** | **15.3** | **0.17** | **7.99** | **Y** | **N** | **α/α** | **α,β/α,β** |
| 85 | 18 | f | N | N | N | N | Y | 265 | <0.10 | <0.10 | 4.56 | N | *^Nd^* | *^Nd^* | *^Nd^* |
| 86 | 49 | f | N | N | N | N | Y | 30 | <0.10 | 0.23 | 6.54 | N | N | α/α | α,β/α,β |
| 87 | 48 | f | N | N | N | N | N | 129 | <0.10 | <0.10 | 6.55 | N | N | α/β | α,β/β,β |
| 88 | 38 | f | N | N | N | N | N | 339 | 0.25 | 18.8 | 4.35 | N | *^Nd^* | *^Nd^* | *^Nd^* |
| 89 | **70** | **f** | **N** | **N** | **N** | **N** | **N** | **49** | **0.64** | **<0.10** | **3.90** | **Y** | ***^Nd^*** | ***^Nd^*** | ***^Nd^*** |
| 90 | 46 | f | **Y** | N | N | N | N | 131 | 1.56 | 0.34 | 3.96 | N | *^Nd^* | *^Nd^* | *^Nd^* |
| 91 | 39 | m | **Y** | N | N | N | N | 417 | 0.85 | 100.0 | 4.74 | N | *^Nd^* | *^Nd^* | *^Nd^* |
| 92 | 60 | m | N | **Y** | **Y** | N | N | 780 | <0.10 | 5.51 | 6.48 | N | N | *^Nd^* | *^Nd^* |

***Note*:** Rows with 14 patients who had *KIT* p.D816V and HαT are highlighted in blue and bold.

^a^Reaction severity grades in HVA were assigned according to the Mueller grading system (grades I−IV).

^b^Tryptase genotyping for HαT was performed in individuals with BST ≥6.0 ng/mL.

***Abbreviations:*** *BST*, basal serum tryptase; *ColdA*, cold-induced anaphylaxis; *ColdA^Cardio^,* cold-induced anaphylaxis with cardiac involvement; *ColdU*, cold urticaria; *f*, female; *HαT*, hereditary α-tryptasemia; *KIT* *p.D816V*, *KIT* missense variant at codon 816 detected in blood leukocytes; *m*, male; *n*, number of patients; *N*, no/negative; *nd*, not determined; *sIgE*, specific immunoglobulin E antibodies; *Y*, yes.
