# Supplementary material for: Cold-induced anaphylaxis: new insights into clinical and genetic characteristics
Source: Front Immunol. 2025 Feb 21;16:1558284. doi: 10.3389/fimmu.2025.1558284 (PMC11885499; doi:10.3389/fimmu.2025.1558284)
Supplement: Supplementary file 2 [file Table2.docx]

**Supplementary Table S2.** **Characteristics of ColdU patients stratified by *KIT* p.D816V.**

| Parameter | Total  *n* = 91 | *KIT* p.D816V  *n* = 6 (6.5) | No *KIT* p.D816V  *n* = 85 (92.4) | *p*-value |
| --- | --- | --- | --- | --- |
| Demographics and baseline characteristics |  |  |  |  |
| Age (years)^a^ | 40.6 ± 13.7 | 49.5 ± 15.7 | 40.0 ± 13.4 | 0.100 |
| Female gender^b^ | 63 (69.2) | 3 (50.0) | 60 (70.6) | 0.367 |
| Duration of ColdU (months)^c^ | 60.0 (14.0−120.0) | 60.0 (24.8−129.0) | 60.0 (14.0−126.0) | 0.885 |
| Age at onset of ColdU (years)^c^ | 33.0 (20.0−42.0) | 47.0 (28.3−54.0) | 33.0 (20.0−41.0) | 0.074 |
| Pediatric onset of ColdU (<18 years)^b^ | 14 (15.4) | 0 | 14 (16.5) | 0.585 |
| Positive family history of ColdU^b^ | 5 (5.5) | 0 | 5 (5.9) | 1.000 |
| Clinical phenotypes |  |  |  |  |
| Typical ColdU^b^ | 48 (52.7) | 2 (33.3) | 46 (54.1) | 0.416 |
| Localized cold-reflex urticaria^b^ | 5 (5.5) | 1 (16.7) | 4 (4.7) | 0.295 |
| ColdU with negative sCST^b^ | 38 (41.8) | 3 (50.0) | 35 (41.2) | 0.691 |
| ColdA | 32 (35.2) | 2 (33.3) | 30 (35.3) | 1.000 |
| ColdA^Cardio^ | 24 (26.4) | 1 (16.7) | 23 (27.1) | 1.000 |
| BST |  |  |  |  |
| BST level (ng/mL)^a^ | 4.67 (3.42−6.48) | 10.87 (7.90−24.20) | 4.56 (3.39−6.04) | **<0.001***** |
| Elevated BST level (>11.4 ng/mL)^b^ | 9 (9.9) | 3 (50.0) | 6 (7.1) | **0.012*** |
| Elevated BST level (>15.0 ng/mL)^b^ | 4 (4.4) | 2 (33.3) | 2 (2.4) | **0.021*** |

***Note*:** Categorical variables are presented as counts (percentages), while numerical variables are expressed as mean ± SD for normally distributed data and median (IQR) for non-normally distributed data. Statistical significance of differences between groups was assessed using the Student’s *t*-test (^a^), Fisher's Exact test (^b^), and Mann-Whitney test (^c^). Statistically significant *p*-values are highlighted in bold. Significance levels are indicated by ***** (*p* < 0.05) and ******* (*p* < 0.001).

***Abbreviations*:** *BST*, basal serum tryptase; *ColdA*, cold-induced anaphylaxis; *ColdA^Cardio^,* cold-induced anaphylaxis with cardiac involvement; *ColdU*, cold urticaria; *KIT p.D816V, KIT* p.D816V missense variant at codon 816 detected in blood leukocytes; *sCST*, standard cold stimulation testing.
