# Supplementary material for: Cold-induced anaphylaxis: new insights into clinical and genetic characteristics
Source: Front Immunol. 2025 Feb 21;16:1558284. doi: 10.3389/fimmu.2025.1558284 (PMC11885499; doi:10.3389/fimmu.2025.1558284)
Supplement: Supplementary file 3 [file Table3.docx]

**Supplementary Table S3. Characteristics of ColdU patients stratified by HαT.**

| Parameter | Total  *n* = 92 | HαT  *n* = 10 (10.9) | No HαT  *n* = 82 (89.1) | *p*-value |
| --- | --- | --- | --- | --- |
| Demographics and baseline characteristics |  |  |  |  |
| Age (years)^a^ | 40.4 ± 13.7 | 43.0 ± 14.0 | 40.1 ± 13.8 | 0.533 |
| Female gender^b^ | 64 (69.6) | 8 (80.0) | 56 (68.3) | 0.718 |
| Duration of ColdU (months)^c^ | 60.0 (14.3−129.0) | 31.5 (9.8−135.0) | 60.0 (15.0−135.0) | 0.518 |
| Age at onset of ColdU (years)^c^ | 33.0 (20.0−42.0) | 33.0 (19.8−46.3) | 33.0 (20.0−42.0) | 0.716 |
| Pediatric onset of ColdU (<18 years)^b^ | 15 (16.3) | 1 (10.0) | 14 (17.1) | 1.000 |
| Positive family history of ColdU^b^ | 5 (5.4) | 1 (10.0) | 4 (4.9) | 0.445 |
| Clinical phenotypes |  |  |  |  |
| Typical ColdU^b^ | 48 (52.2) | 6 (60.0) | 42 (51.2) | 0.742 |
| Localized cold-reflex urticaria^b^ | 5 (5.4) | 0 | 5 (6.1) | 1.000 |
| ColdU with negative sCST^b^ | 39 (42.4) | 4 (40.0) | 35 (42.7) | 1.000 |
| ColdA | 33 (35.9) | 5 (50.0) | 28 (34.1) | 0.486 |
| ColdA^Cardio^ | 25 (27.2) | 3 (30.0) | 22 (26.8) | 1.000 |
| BST |  |  |  |  |
| BST level (ng/mL)^a^ | 4.70 (3.46−6.45) | 13.40 (11.48−16.20) | 4.50 (3.34−5.84) | **<0.001***** |
| Elevated BST level (>11.4 ng/mL)^b^ | 9 (9.8) | 8 (80.0) | 1 (1.2) | **<0.001***** |
| Elevated BST level (>15.0 ng/mL)^b^ | 4 (4.3) | 3 (30.0) | 1 (1.2) | **0.004**** |

***Note*:** Categorical variables are presented as counts (percentages), while numerical variables are expressed as mean ± SD for normally distributed data and median (IQR) for non-normally distributed data. Statistical significance of differences between groups was assessed using the Student’s *t*-test (^a^), Fisher's Exact test (^b^) and Mann-Whitney test (^c^). Statistically significant *p*-values are highlighted in bold. Significance levels are indicated by ****** (*p* < 0.01) and ******* (*p* < 0.001).

***Abbreviations*:** *BST*, basal serum tryptase; *ColdA*, cold-induced anaphylaxis; *ColdA^Cardio^,* cold-induced anaphylaxis with cardiac involvement; *ColdU*, cold urticaria; *HαT*, hereditary α-tryptasemia; *sCST*, standard cold stimulation testing.
